# Excess body weight in the city of São Paulo: panorama from 2003 to 2015, associated factors and projection for the next years

**DOI:** 10.1186/s12889-018-6225-8

**Published:** 2018-12-03

**Authors:** Jaqueline Lopes Pereira, Diva Aliete dos Santos Vieira, Maria Cecília Goi Porto Alves, Chester Luís Galvão César, Moisés Goldbaum, Regina Mara Fisberg

**Affiliations:** 1 Department of Nutrition, School of Public Health, University of São Paulo, São Paulo, Brazil; 2 Department of Nutrition, Federal University of Sergipe, Lagarto, Brazil; 3 Department of Health of the State of São Paulo, Institut of Health, São Paulo, SP Brazil; 4 Department of Epidemiology, School of Public Health, University of São Paulo, São Paulo, Brazil; 5 Department of Preventive Medicine, Medical School, University of São Paulo, São Paulo, Brazil

**Keywords:** Overweight, Obesity, Survey, Population, São Paulo, Brazil

## Abstract

**Background:**

Excess body weight (EBW: overweight and obesity) has high and rising prevalence in Brazil. Up-to-date information about the distribution and changes in the prevalence of EBW and their associated factors are essential to determine target groups and to identify priority actions. The aim of this study was to investigate the associated factors and to determine the prevalence of overweight and obesity in the adolescent and adult population of the city of São Paulo in the years of 2003, 2008, and 2015, as well as to estimate the prediction for the next years.

**Methods:**

Individuals aged 12 years and older from three editions of the Health Survey of São Paulo (ISA-Capital), a cross-sectional population-based survey, carried out in 2003 (*n* = 2144), 2008 (*n* = 2599), and 2015 (*n* = 3939), had their socioeconomic, anthropometric, and lifestyle data collected at households. Individuals were classified according to their age and BMI as: without excess body weight, overweight, or obese. Differences were evaluated through Pearson’s Chi-square test and comparison of 95% CI. Generalized ordered logit models were used to evaluate factors associated to overweight/obesity and logistic regression models were used to predict their prevalence for the next years.

**Results:**

The prevalence (95% CI) of obesity in total population doubled: from 10% (8.0, 12.5) in 2003 to 19.2% (17.8, 20.6) in 2015. The main increase occurred in female adolescents from 2.5% (1.2, 5.3) to 11.2% (8.4, 14.7) and adults, from 9.2% (6.4, 13.1) to 22.3% (20.0, 24.8). Those with higher chance of having EBW were adults, those with higher income, and former smokers. The prevalence of EBW increased 31% from 2003 to 2008, and 126% from 2003 to 2015, when half of the population had EBW. If this pattern does not change, 77% of the population is expected to have EBW by 2030.

**Conclusions:**

Our findings present up-to-date information about the distribution of EBW, which increased substantially over a short time and more prominently in specific groups. The factors associated with EBW may provide important information for decision makers and researchers to create or review the existing programs and interventions in order to decrease the trend for the next years.

## Background

In the last decades, excess body weight, including overweight and obesity, has gained importance worldwide because of the high and rising prevalence in many countries, with variations in the levels and trends according to specific regional patterns [1]. Worldwide, the prevalence of excess body weight between 1980 and 2013 rose by 27.5% for adults and 47.1% for children [1]. In 2016, excess body weight was present in 340 million children and adolescents aged 5–19 and in more than 1.9 billion adults (39%); of these over 650 million (13%) were obese [2]. More than 50% of adult population of men in Tonga and of women in Kuwait, Kiribati, Federated States of Micronesia, Libya, Qatar, Tonga, and Samoa are obese. Regardless of developed countries have attenuated the increase in obesity in the last decade, many of them still have a very high prevalence, and it is continually increasing in developing countries, where almost two in three obese people in the world live [1].

This is a public health concern because overweight and obesity are important risk factors for other diseases with high morbidity and mortality rates, such as diabetes, some types of cancer, cardiovascular, respiratory and musculoskeletal diseases [3]. A systematic evaluation of the health effects of high body mass index (BMI) estimated that excess body weight accounted for about 4 million deaths and 120 million disability-adjusted life-years worldwide in 2015 [4]. Thus, besides individual health problems, excess body weight also causes a significant increase in direct and indirect health costs, becoming a problem for the economy and the health systems [5–7]. A study with the 2008–2009 Brazilian Household Budget Survey estimated that the presence of an obese individual in the household was associated with 19% higher monthly expenses on medicines per capita compared to households without an obese resident [6]. The estimated direct costs associated to patient care in one year in the Brazilian Health System (SUS) with all diseases related to excess body weight exceed US$ 2.1 billion, and approximately 10% of this value is attributable exclusively to overweight and obesity [7].

Up-to-date information about the distribution, magnitude and changes in the prevalence of excess body weight across the years as well as their associated factors are essential to help decision makers and researchers to determine target groups and to identify priority actions for interventions to decrease excess body weight in the population [1, 8]. In this context, studies have reported associations between excess body weight and factors such as age, gender, socioeconomic status, physical activity, tobacco and alcohol intake, diet quality and several others, that may differ in the strength and direction of this relationship according to each population [1, 9–14].

Nationally representative survey data show that the prevalence of excess body weight in Brazil has steadily increased over the past four decades, with disparities between population groups [15, 16]. According to the Surveillance of Risk and Protective Factors for Chronic Diseases by Telephone Survey, VIGITEL [17], more than half of the population has excess body weight, wherein one in every five Brazilians is obese. This national survey indicates that in São Paulo, which is the biggest city in Brazil and one of the most populous cities in the world, with more than 12 million habitants [18], the outlook is similar to the country: 54% of adults have excess body weight and 18% are obese [17]. Although VIGITEL monitors the frequency and distribution of the main determinants of chronic noncommunicable diseases (NCD), such as smoking status, alcohol intake, and physical activity in the 26 State capitals and Federal District, the survey does not explore the direct association of these factors with the body weight status. Also, because it is a survey design to get the information by telephone interviews, it allows inference for the population who lives in households with landline telephone. In addition, the survey sample includes people aged 18 years and older. In Brazil, 17% of adolescents are overweight and 8% are obese, and this is an important age group, especially regarding opportunities for prevention of excess body weight related diseases in future life [19, 20].

In this context, the Health Survey of São Paulo (*Inquérito de Saúde de São Paulo*, ISA-Capital) is a cross-sectional population-based survey, conducted periodically to evaluate the health status and the use of health services of a probabilistic sample of individuals aged 12 years and older living in the city of São Paulo [21]. With face-to-face interviews carried out in the households, the ISA-Capital provides detailed information regarding socioeconomic and lifestyle characteristics, which allows a current overview of the health in the city. The prevalence of excess body weight in the city was previously published in the Municipal Government Report [22], however the associated factors were not investigated. Therefore, the aim of this study is to determine the prevalence of overweight and obesity in the adolescent and adult population of the city of São Paulo in the years of 2003, 2008, and 2015, to investigate the associated factors as well as to estimate the prediction for the next years. The concept underlying our hypothesis is that the sociodemographic factors evaluated in this survey will be dissimilarly associated to excess body weight in this population.

## Methods

### Population and study design

The present paper analyses data from three editions of the Health Survey of São Paulo (ISA-Capital), a cross-sectional population-based survey that aimed to evaluate the health status and the use of health services in a representative sample of residents of the city of São Paulo, Southeastern Brazil. The surveys were carried out in 3 years: 2003 (*n* = 3357), 2008 (*n* = 3271), and 2015 (*n* = 4043) and employed a similar sampling process. They used a complex sampling design with a two-stage cluster: census tracts and households. Details of the studies and their sampling design are published elsewhere [23, 24]. The surveys were approved by Ethics Committee on Research of the School of Public Health, University of São Paulo. Written informed consent/assent was obtained before commencement of the study from all subjects and, when adolescent, also from their proxies.

For the present study, inclusion criteria were individuals aged at least 12 years old, with complete anthropometric, age and sex information. We analyzed a total of 2144 individuals (711 adolescents from 12 to 19 years old, 711 adults from 20 to 59 years old, and 722 older adults aged 60 years or more) for the 2003 ISA-Capital; a total of 2599 individuals (569 adolescents, 1141 adults, and 889 older adults) for the 2008 ISA-Capital; and a total of 3939 individuals (822 adolescents, 2126 adults, and 991 older adults) for the 2015 ISA-Capital.

Trained interviewers used a structured questionnaire administered at households to collect individuals demographic (sex, age, race, marital status) and socioeconomic data (family income, educational level, working status), as well as lifestyle information (smoking status, alcohol consumption, and physical activity).

Total household income was assessed adding all the net income (individual’s income after taking taxes and deductions into account) of each individual in the household, including wages, retirement, government benefits, pension, grants, rental income, and any other. The total amount was divided by the number of persons in the household in order to estimate the per capita household income. For analysis purposes, this variable was categorized as ≤1 minimum wage or > 1 minimum wage, in order to enable the comparison across the survey years. One minimum wage is approximately 78 US dollars in 2003, 217 US dollars in 2008, and 236 US dollars in 2015.

Questions about smoking habits, tobacco use and number and frequency of cigarettes per day were performed in order to define smoking status. Those who referred currently smoke at least one cigarette per day, every day, for at least 1 month, were considered smokers. Those who referred not currently smoke, but have already smoked at least one cigarette per day, every day, for at least 1 month in the past were considered former-smokers [25].

Alcohol intake was evaluated in ISA-Capital 2003 and 2008 using the “*Cut Down, Annoyed by Criticism, Guilty and Eyeopener”* (CAGE) [26] and in ISA-Capital 2015, using the “*Alcohol Use Disorders Identification Test*” (AUDIT) [27], associated to complementary questions. We used the frequency of alcohol consumption: never, ≤ 3 times per week, or ≥ 4 times per week, in order to enable the comparison across the individuals from the three surveys.

Physical activity was assessed using the long International Physical Activity Questionnaire, validated for Brazilian population [28]. Individuals were classified as ‘meet the recommendation’ or ‘do not meet the recommendation’ according to the latest recommendations for global physical activity of World Health Organization for each life stage: ≥420 min/week, including 60 min/day for adolescents; ≥150 min/week for adults and older adults [29].

### Anthropometric measurements

Height and weight, used to calculate the body mass index (BMI = weight (kg) / squared height (m^2^)), were self-reported. The use of self-reported high and weight is known to incur in possible errors, but previous study with the same population showed good agreement between measured and self-report weight, height and BMI [30].

Individuals were classified according to their age and BMI into three groups: without excess body weight, overweight, or obese. Adolescents were classified according to the World Health Organization curves for children and adolescents [31], in which they were considered overweight when BMI-for-age was > + 1SD and ≤ +2SD (equivalent to BMI 25 kg/m^2^ at 19 years) and obese when BMI-for-age > +2SD (equivalent to BMI 30 kg/m^2^ at 19 years). Adults were considered overweight when 25 ≤ BMI < 30 kg/m^2^ and obese when BMI ≥ 30 kg/m^2^ [8]. Older adults were classified with overweight (28 < BMI < 30 kg /m^2^) or obesity (BMI ≥ 30 kg /m^2^) according to the cut-off points recommended by the Pan American Health Organization (OPAS) in the Health, Well-Being, and Aging Study (SABE) with Latin American countries, including Brazil [32]. We used the term excess body weight when referring to overweight and obesity combined.

### Statistical analysis

Stata software was used in all analysis (Statistics/Data Analysis, version 13.1, Texas, USA), considering the complex sampling design (*svy* commands). All statistical tests considered the significance level of 5%. Differences in the prevalence of socioeconomic and demographic variables according to ISA-Capital year after running proportion on estimation were evaluated through Pearson’s Chi-square test with the Rao and Scott second-order correction and comparison of the 95% confidence interval (CI).

Generalized ordered logit models for ordinal dependent variables were used to evaluate the factors associated to overweight/obesity (without excess body weight = 0, overweight = 1, obese = 2) [33, 34]. The following variables were statistically significant (*p* < 0.20) in the univariate analysis and were included in the models: sex (male or female), age group (adolescents, adults, or older adults), self-reported race (white or non-white), marital status (married/with partner, single, separated/divorced, or widow(er)), working status (working, not working, student only, other), per capita family income (more or less than one minimum wage), education of householder (more or less than high school), smoking status (non-smoker, former smoker, or current smoker), alcohol consumption (never, ≤ 3 times per week, or ≥ 4 times per week), physical activity level (meet or do not meet the WHO recommendations), and ISA-Capital year (2003, 2008, or 2015). Wald tests were used to test the proportional odds assumption in the model. The final multiple model did not present any negative predicted probabilities and the values of Basic Information Criterion (BIC) and Akaike’s Information Criterion (AIC) were compared in order to choose the more parsimonious and adequate model. The factors were considered associated to overweight or obesity when *p*-value < 0.05.

In order to predict the prevalence of overweight and obesity for the next years, we used logistic regression models [35] with the same variables of the ordered logit models, except marital status (due to the low number of individuals in some categories across the age groups). We conducted two models for each age group: a) without excess body weight = 0, excess body weight = 1; and b) without excess body weight = 0, obese = 1. The coefficients of the models were used to calculate the prevalence for each age group: Prob(y = 1) = 1/ 1 + exp-(β_0_ + β_1_*year).

## Results

The number of adolescents in the population decreased from 2003 to 2015, as well as the proportion of people who declare themselves white, single, families whose householder did not complete high school, and those with higher income. In contrast, there was an increase in the proportion of older adults, non-smokers, people working, and who do not consume alcoholic beverages (Table 1, total population). The prevalence of obesity doubled in the period. In 2015, considering all age groups, 21% of female and 17% of male were obese. The prevalence of overweight and obesity for total population was 26 and 10% in 2003, 29 and 13% in 2008, and 30 and 19% in 2015, respectively. During this time period, the prevalence of both overweight and obesity increased in men, in those who declared themselves non-white, single, working, with lower income and education, and non-consumers of alcohol. Obesity rates increased in adolescents and adults, in women, in those who declared themselves white, married, not working, with higher income and education, non- and former smokers, moderate consumers of alcohol, and in both physical activity levels (Table 1).

**Table 1:** Characteristics of subjects in the Health Survey of São Paulo (ISA-Capital) 2003, 2008, and 2015, for total population and for overweight and obesity (*n* = 8682)

	ISA-Capital 2003 (n = 2144)							ISA-Capital 2008 (n = 2599)						
	Total population		Overweight		Obese			Total population		Overweight		Obese		
	%	95%CI	%	95%CI	%	95%CI	p^a^	%	95%CI	%	95%CI	%	95%CI	p^a^
*Age group*														
Adolescents (12–19 yrs)	17.9	16.1, 19.8	16.2	12.6, 20.6	3.7	2.3, 5.9		14.7	12.9, 16.7	19.5	16.1, 23.3	5.9	4.1, 8.5	
Adults (20–59 yrs)	69.7	67.7, 71.6	30.9	27.1, 35.0	10.6	8.1, 13.9		71.6	69.0, 74.0	34.4	31.5, 37.4	13.2	11.2, 15.5	
Older adults (≥60 yrs)	12.5	10.9, 14.2	11.5	9.2, 14.2	15.7	12.5, 19.6	< 0.0001	13.7	11.8, 16.0	13.7	11.7, 16.0	19.1	16.0, 22.5	< 0.0001
*Sex*														
Male	47.7	45.0, 50.5	27.3	23.4, 31.7	11.0	8.3, 14.4		47.2	45.2, 49.2	33.6	30.1, 37.3	12.2	9.8, 15.1	
Female	52.3	49.5, 55.0	24.5	20.6, 28.9	9.2	7.0, 11.9	0.2602	52.8	50.8, 54.8	25.6	23.2, 28.0	13.6	11.6, 15.8	0.0012
*Race/Skin color*														
White	67.2	63.2, 70.9	27.3	24.0, 31.0	10.7	8.1, 14.1		62.0	56.9, 66.8	30.2	27.5, 33.0	13.2	11.2, 15.6	
Non white	32.8	29.1, 36.8	22.7	18.8, 27.2	8.7	6.4, 11.9	0.0832	38.0	33.2, 43.1	27.8	24.3, 31.6	12.4	10.5, 14.7	0.3851
*Marital status*														
Married / partners	39.1	35.5, 42.9	31.7	24.8, 39.6	10.9	7.2, 16.1		42.7	39.8, 45.6	33.5	29.6, 37.6	15.1	12.0, 18.8	
Single	56.8	53.1, 60.5	17.1	13.7, 21.2	5.3	2.9, 9.6		51.7	48.6, 54.7	19.6	16.6, 23.0	6.6	4.8, 8.8	
Separated / Divorced	1.9	1.2, 3.1	28.6	10.6, 57.5	8.4	1.5, 34.9		3.5	2.6, 4.7	17.3	8.4, 32.4	13.4	6.1, 27.0	
Widow(er)	2.2	1.6, 3.0	7.3	3.3, 15.6	13.5	5.3, 30.2	< 0.0001	2.2	1.6, 2.9	8.4	3.6, 18.2	19.5	10.5, 33.2	< 0.0001
*Working status*														
Working	45.1	40.6, 49.8	21.6	17.0, 27.0	8.5	5.8, 12.3		53.4	49.7, 57.1	29.6	25.6, 33.9	9.1	6.6, 12.3	
Not working	29.5	26.4, 32.8	30.1	21.7, 40.0	9.7	5.6, 16.3		26.7	21.7, 27.9	21.2	16.8, 26.2	17.4	13.4, 22.3	
Student only	22.6	19.4, 26.1	17.7	13.7, 22.7	5.2	2.2, 11.6		20.8	17.9, 24.0	19.8	15.7, 24.6	7.1	4.3, 11.4	
Other	2.9	1.9, 4.4	10.8	3.9, 26.5	0.0	–	0.0197	1.1	0.7, 1.9	5.1	1.1, 19.8	10.5	2.9, 31.4	< 0.0001
Per capita *family income*														
≤ 1 minimum wage	35.1	30.8, 39.7	22.1	18.3, 26.4	7.8	5.5, 10.8		36.4	31.2, 41.9	25.5	22.1. 29.3	12.6	10.3, 15.3	
> 1 minimum wage	64.9	60.3, 69.3	27.2	23.4, 31.3	11.0	8.2, 14.7	0.0339	63.7	58.1, 68.9	31.7	28.9, 34.6	13.1	11.0, 15.6	0.0213
*Education of householder*														
Less than High School	54.0	49.3, 58.6	23.7	20.5, 27.3	10.3	8.0, 13.2		43.6	37.1, 50.4	26.6	23.6, 30.0	13.5	11.5, 15.9	
High School or higher	46.0	41.5, 50.7	28.2	23.9, 32.9	9.6	6.6, 13.8	0.2958	56.4	49.6, 62.9	31.7	28.8, 34.8	12.3	9.8, 15.2	0.0963
*Smoking status*														
Non-smoker	65.5	62.7, 68.2	25.1	21.7, 28.9	9.9	7.7, 12.6		64.3	61.5, 67.1	28.4	25.4, 31.5	11.4	9.6, 13.4	
Former smoker	15.2	12.8, 18.0	35.7	28.8, 43.2	13.5	8.8, 20.3		16.4	14.4, 18.7	33.5	29.0, 38.4	21.3	17.3, 25.8	
Current smoker	19.3	16.8, 22.0	20.4	15.1, 27.0	8.2	4.5, 14.3	0.0037	19.2	16.9, 21.9	29.1	24.6, 34.1	10.9	7.6, 15.5	< 0.0001
*Alcohol intake*														
Never	46.2	42.6, 49.8	22.4	19.2, 25.9	11.2	8.8, 14.1		43.6	40.8, 46.4	25.9	22.8, 29.1	12.7	10.4, 15.3	
≤ 3 times per week	48.4	44.8, 51.9	28.1	24.0, 32.5	9.2	6.6, 12.7		51.0	48.3, 53.7	32.4	29.4, 35.7	13.0	10.7, 15.9	
≥ 4 times per week	5.5	4.2, 7.1	36.2	25.4, 48.7	9.4	4.2, 19.5	0.0460	5.5	4.4, 6.8	29.8	22.3, 38.6	13.2	8.2, 20.8	0.0427
*Physical activity level*														
Do not meet	22.8	19.9, 26.1	24.2	19.4, 29.7	11.6	8.3, 16.0		19.3	17.1, 21.8	22.9	18.5, 27.8	13.1	9.8, 17.2	
Meet the recommendations	77.2	73.9, 80.1	26.2	23.0, 29.6	9.6	7.5, 12.2	0.5079	80.7	78.2, 82.9	30.9	28.5, 33.4	12.9	11.0, 15.0	0.0224
*Body weight status*														
Overweight	25.9	23.1, 28.8	–	–	–	–		29.4	27.3, 31.6	–	–	–	–	
Obesity	10.0	8.0, 12.5	–	–	–	–		12.9	11.3, 14.7	–	–	–	–	
	ISA-Capital 2015 (n = 3939)													
	Total population		Overweight		Obese									
	%	95%CI	%	95%CI	%	95%CI	p^a^	p^b^						
*Age group*														
Adolescents (12–19 yrs)	13.2	12.0, 14.4	19.5	16.9, 22.4	9.3	7.6, 11.4								
Adults (20–59 yrs)	70.8	69.1, 72.5	36.2	34.0, 38.4	20.5	18.7, 22.5								
Older adults (≥60 yrs)	16.0	14.3, 17.9	13.2	10.9, 15.9	21.2	18.7, 24.0	< 0.0001	0.0008						
*Sex*														
Male	47.0	45.4, 48.6	34.4	31.8, 37.1	17.1	14.9, 19.5								
Female	53.0	51.4, 54.6	26.7	24.4, 29.1	21.0	19.2, 23.0	< 0.0001	0.8798						
*Race/Skin color*														
White	51.4	48.0, 54.8	30.4	27.8, 33.1	19.6	17.5, 21.9								
Non white	48.6	45.2, 52.0	30.0	27.9, 32.2	18.9	17.2, 20.7	0.8032	< 0.0001						
*Marital status*														
Married / partners	51.7	49.5, 53.9	34.0	31.4, 36.7	23.4	21.1, 25.8								
Single	35.2	33.3, 37.1	25.6	23.2, 28.3	12.0	10.2, 14.1								
Separated / Divorced	7.6	6.7, 8.7	32.9	27.5, 38.8	21.1	16.4, 26.7								
Widow(er)	5.5	4.8, 6.3	19.6	15.0, 25.3	23.4	18.8, 28.7	< 0.0001	< 0.0001						
*Working status*														
Working	60.6	58.6, 62.7	34.8	32.4, 37.3	19.6	17.6, 21.7								
Not working	27.6	25.7, 29.5	24.6	21.8, 27.6	22.1	19.3, 25.2								
Student only	10.8	9.7, 12.0	19.0	16.0, 22.4	10.1	7.7, 13.2								
Other	1.0	0.7, 1.4	27.6	15.5, 44.3	16.0	7.8, 30.2	< 0.0001	< 0.0001						
Per capita *family income*														
≤ 1 minimum wage	49.0	45.0, 53.0	30.7	28.4, 33.1	19.1	16.8, 21.5								
> 1 minimum wage	51.0	47.0, 55.0	32.1	28.8, 35.6	19.5	17.1, 22.1	0.7034	0.0059						
*Education of householder*														
Less than High School	43.0	39.3, 46.9	29.9	27.6, 32.2	19.3	17.5, 21.4								
High School or higher	57.0	53.1, 60.7	30.8	28.3, 33.4	19.5	17.3, 21.8	0.8300	0.0083						
*Smoking status*														
Non-smoker	70.3	68.5, 72.0	29.4	27.3, 31.5	18.5	16.9, 20.3								
Former smoker	13.6	12.3, 15.1	38.0	33.6, 42.6	24.1	20.5, 28.0								
Current smoker	16.1	14.8, 17.6	28.1	24.6, 32.0	17.7	14.0, 22.2	< 0.0001	0.0159						
*Alcohol intake*														
Never	62.7	62.9, 68.4	27.9	26.1, 29.8	19.4	17.7, 21.2								
≤ 3 times per week	30.9	28.5, 33.5	34.9	31.4, 38.5	19.0	16.0, 22.4								
≥ 4 times per week	3.4	2.8, 4.2	30.7	21.4, 41.8	11.3	6.4, 19.2	0.0042	< 0.0001						
*Physical activity level*														
Do not meet	21.9	19.8, 24.1	25.4	22.7, 28.4	20.4	17.8, 23.2								
Meet the recommendations	78.1	75.9, 80.2	31.6	29.5, 33.8	18.7	17.2, 20.3	0.0028	0.3880						
*Body weight status*														
Overweight	30.3	28.6, 32.1	–	–	–	–								
Obesity	19.2	17.8, 20.6	–	–	–	–		< 0.0001						

^a^Pearson’s Chi-square test with the Rao and Scott second-order correction *p*-values for differences in each ISA-Capital

^b^Pearson’s Chi-square test with the Rao and Scott second-order correction *p*-values for differences in total population prevalence across ISA-Capital surveys

**Fig. 1:**
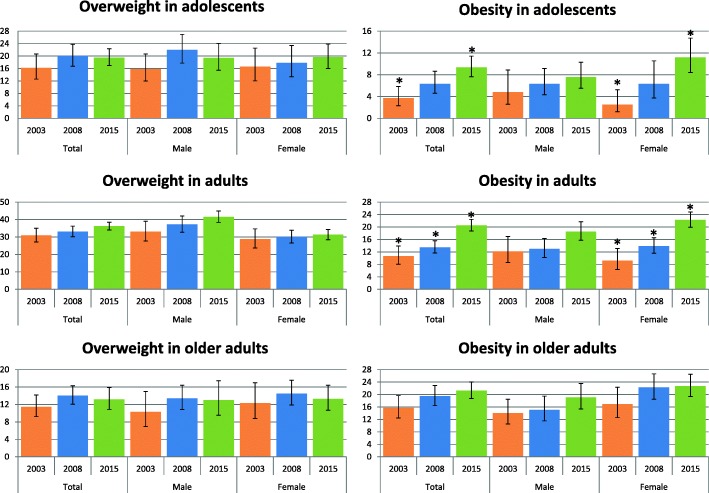
Prevalence of overweight and obesity according to age and sex in the population of Sao Paulo. ISA-Capital 2003–2008 - 2015

Figure 1 presents the prevalence of overweight and obesity in the population of São Paulo according to age group in 2003, 2008 and 2015. Obesity increased in adolescents from 3.7% in 2003 to 9.3% in 2015 (in girls the prevalence increased from 2.5 to 11.2%) and it doubled in adults in this period (in women it was 9.2% in 2003 and 22.3% in 2015). Despite an apparent increase in the prevalence of overweight in adults and obesity in older adults, the difference was not significant. In 2015, the prevalence of overweight according to gender was similar in adolescents (19%) and older adults (13%), but it was higher in men (41.6%) than in women (31.3%) in adult population (20–59 years old).

Table 2 shows the results of the multivariable logistic regression analysis. Excess body weight and obesity were associated with age group, sex, race, marital status, income, smoking status, physical activity level and ISA-Capital year. Adults were 67% more likely to have excess body weight or obesity compared to adolescents. Older adults were 43% less likely to have excess body weight, but 60% more likely to be obese then adolescents. That is, adolescents were more likely to be overweight, while older adults were more likely to be obese.

**Table 2:** Odds ratios of ordered logistic regression on overweight and obesity in the Health Survey of São Paulo (ISA-Capital) 2003, 2008, and 2015^a^

	Overweight & Obese vs Not overweight/obese		Obese vs (overweight or not overweight/obese)	
	Total population		Total population	
	OR	95%CI	%	95%CI
*Age group (ref. Adolescents)*				
Adults (20–59 years)	1.67**	1.30, 2.13		Same^a^
Older adults (60 years or more)	0.57**	0.43, 0.75	1.60**	1.21, 2.12
*Sex (ref. Male)*				
Female	0.80**	0.71, 0.90	1.10	0.94, 1.28
*Race/Skin color (ref. White)*				
Non white	0.84*	0.76, 0.94	0.99	0.86, 1.13
*Marital status (ref. Married / Common law partners)*				
Single	0.43**	0.36, 0.51	0.51**	0.41, 0.63
Separated / Divorced	0.81	0.64, 1.04		Same^a^
Widow(er)	0.96	0.74, 1.24		Same^a^
*Working status (ref. Working)*				
Not working	1.11	0.93, 1.31		Same^a^
Student only	1.08	0.81, 1.45		Same^a^
Other	0.70	0.42, 1.17		Same^a^
Per capita *family income (ref. ≤ 1 minimum wage)*				
> 1 minimum wage	1.14*	1.00, 1.30		Same^a^
*Education of householder (ref. Less than High School)*				
High School or higher	0.91	0.81, 1.02		Same^a^
*Smoking status (ref. Non-smoker)*				
Former smoker	1.40**	1.21, 1.63		Same^a^
Current smoker	0.67**	0.57, 0.79	0.83	0.66, 1.03
*Alcohol intake (ref. Never)*				
≤ 3 times per week	1.06	0.93, 1.20		Same^a^
≥ 4 times per week	0.90	0.70, 1.16		Same^a^
*Physical activity level (ref. Do not meet the recommendations)*				
Meet the recommendations	0.91	0.79, 1.05	0.76*	0.65, 0.89
*Year ISA-Capital (ref. 2003)*				
2008	1.32**	1.12, 1.52		Same^a^
2015	2.20**	1.81, 2.68		Same^a^

^a^The multiple model was adjusted by age group, sex, self-reported race, marital status, working status, per capita family income, education of householder, smoking status, alcohol consumption, physical activity level, and ISA-Capital year

^b^Estimates for Overweight & Obese vs Not overweight/obese are the same as for Obese vs (overweight or not overweight/obese)

* *p* < 0.05 **p < 0.005

Despite women being 20% less likely to have excess body weight than men, the association was inverse, but not significant, when only obesity was evaluated. Those who declared themselves white or single presented lower odds ratio for both excess body weight and obesity; and those with higher income and former smokers presented higher odds ratio compared to their counterparts. Current smokers were less likely to have excess body weight. Also, those who meet the physical activity level recommendation were 24% less likely to be obese compared to those who do not meet the recommendation.

**Fig. 2:**
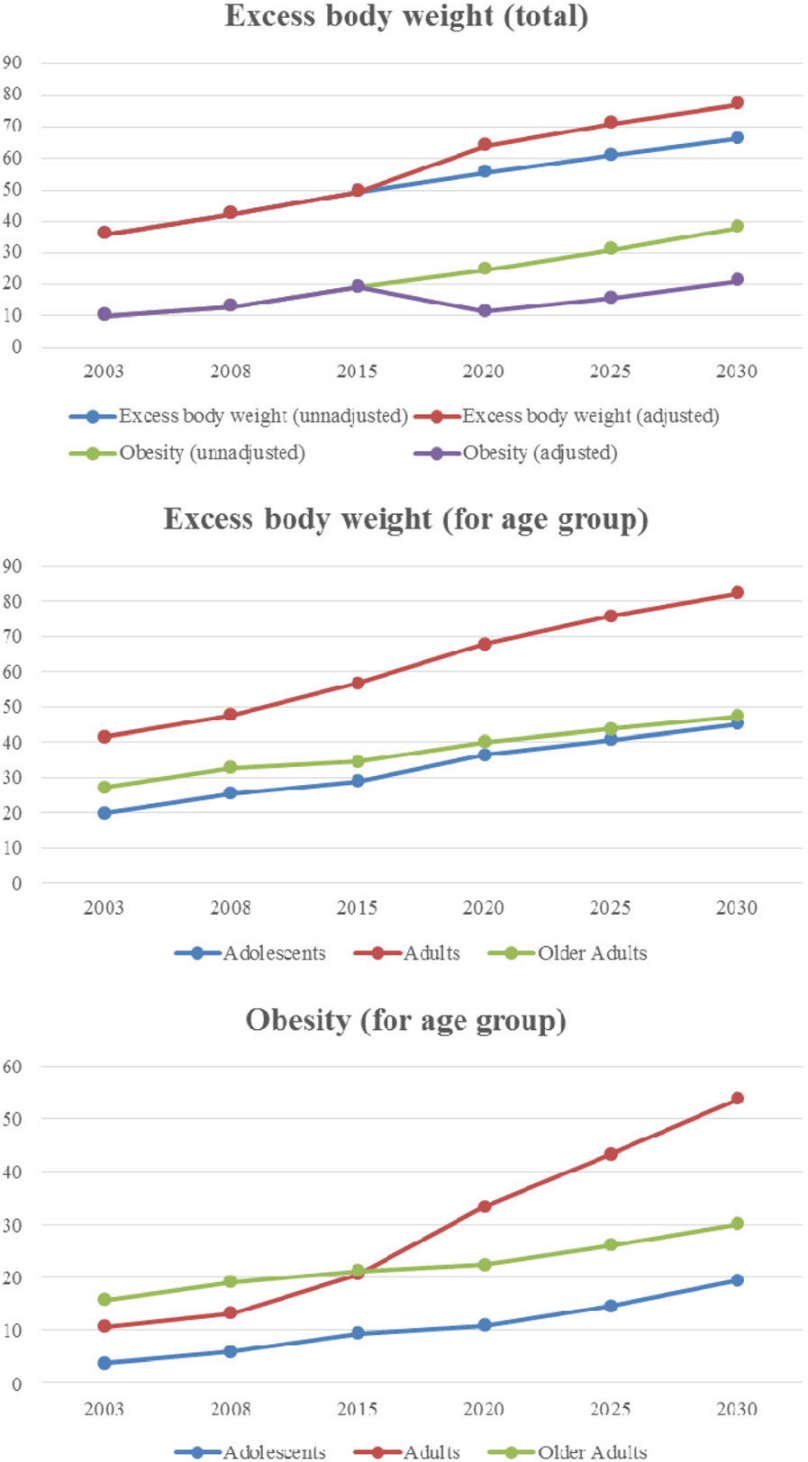
Prevalence of excess body weight and obesity predicted by logistic regression models for the population of Sao Paulo according to age group for the years of 2020, 2025 e 2030. ISA-Capital

An important increase in both excess body weight and obesity occurred across ISA-Capital years (OR = 1.3 in 2008 and OR = 2.2 in 2015). Due to this alarming growth, the prevalence of excess body weight and obesity was predicted using logistic regression models, considering the scenario observed in the last surveys. The models were adjusted by the same variables of the model presented in Table 2. Figure 2 illustrates the projection for the years of 2020, 2025, and 2030, when 77% of the population is expected to have excess body weight if the scenario continues the same observed in previous surveys. By 2030, the prevalence of obesity is likely to be 19% for adolescents, 54% for adults, and 30% for older adults.

## Discussion

Excess body weight, particularly obesity, increased in the population of Sao Paulo from 2003 to 2015, especially in female adolescents and adults. In general, the prevalence increased 31% from 2003 to 2008, and 126% from 2003 to 2015. If this pattern does not change, more than three-quarters of the population will have excess body weight by 2030. Those with higher chance of having both overweight and obesity were adults, those with higher income, and former smokers.

The prevalence of obesity for the total population in ISA-Capital in 2015 (19.2, 95% CI = 17.8, 20.6%) was similar to the observed in Sao Paulo in VIGITEL 2016 (18.1, 95% CI = 16.2, 20.0%) [17], despite the aforementioned differences between the studies designs: the telephone interviews in people aged 18 years old or more in VIGITEL versus the household interviews in people aged 12 years old or more in ISA-Capital. The frequency of obesity is comparable to other Brazilian State capitals with diverse populations and characteristics, such as Curitiba (18.9%), Boa Vista (18.7%), and Macapá (17.7%) [17]. However, these cities present higher prevalence of both excess body weight and obesity in men, while in Sao Paulo men have higher rates of excess body weight while women have higher rates of obesity. This pattern is consistent with the observed in developed countries over time [1]. The observed differences across the cities suggest that environmental factors, such as urbanization, physical, economic and social contexts, and food environment [10, 36–38], could play an important role regarding sex differences in obesity.

The prevalence of obesity in the city of São Paulo in 2015 among female adolescents (11.2%; 95% CI: 8.4, 14.7%) is similar to countries such as Australia (11.2%; 95% CI: 6.4, 17.0%), Turkey (10.9%; 95% CI: 4.0, 20.8%), and Uruguay (11.5%; 95% CI: 3.4, 23.1%) and among male adolescents (7.6%; 95% CI: 5.5, 10.3%), it is similar to Colombia (7.5%; 95% CI: 3.0, 14.0%), Switzerland (7.0%; 95% CI: 3.5, 11.8%), and Kazakhstan (7.8%; 95% CI: 1.5, 19.0%) [39]. The prevalence among adult women (22.3%; 95% CI: 19.9, 24.8%) is similar to the observed in European countries such as France (22%; 95% CI: 16.2, 28.3%), Portugal (22.1%; 95% CI: 16.3, 28.4%), Spain (23.8%; 95% CI: 18.7, 29.0%), and Romania (22.5%; 95% CI: 16.7, 29.1%) [39]. Among adult men (18.6%; 95% CI: 15.8, 21.7%), the prevalence is comparable to Colombia (18.3%; 95% CI: 12.9, 24.2%), Panama (18.5%; 95% CI: 12.5, 25.5%), and Russia (18.9%; 95% CI: 14.0, 24.5%) [39]. Among older adults, the prevalence of obesity is similar to the observed in Austria (21.3%), Belgium (20.4%), France (20.8%), and Spain (20.9%), considering both sex [40].

In 2015, 30% of the population of Sao Paulo was overweight, with the highest prevalence for adults (36%), especially men (42%), similar to countries such as Spain (42.1%), Germany (42.4%), and Portugal (42.9%) [1]. Although a lot of attention is given to obesity, overweight is also an important public health issue, since the risk of death continuously increases for adults with BMI above 25 kg/m^2^. A study of 67.8 million individuals worldwide showed that 40% of deaths and 38% of the disability-adjusted life years (DALYs) related to high BMI occurred among non-obese individuals, indicating that a significant proportion of the total burden would be missed by focusing exclusively on obese individuals [4]. In addition, since weight gain is usually a gradual process caused by small changes in energy balance over time, for individuals to become obese they must first be overweight during a period of their lives, and this time may be a good opportunity for prevention [41, 42].

The younger population is also a significant target for prevention. Children and adolescents with excess body weight are more likely to have several health problems in their present and future lives, such as chronic diseases (e.g., asthma), cardiovascular risk factors (e.g., high blood pressure), and poor mental health (e.g., low self-esteem) [20, 41]. Obese adolescents are five times more likely to become obese adults, which increases the potential for morbidity and premature mortality across the life [43]. In the present study, the prevalence of overweight in adolescents from 12 to 19 years old was 19% and they were 43% more likely to have excess body weight compared to older adults. In spite of the lack of change in overweight prevalence across the years, obesity increased significantly, especially in girls. Studies conducted in Brazil observed that, compared to boys, girls are less physically active [44], have more sedentary leisure time [45], skip breakfast more frequently [46], and consume more sugar and sweet food [47], besides the sex disparities in fat metabolism, fat storage, and puberty [48, 49], which are possible explanations for the observed differences.

Another important factor associated with both excess body weight and obesity in the present study was smoking status. Similar to our findings, a study with 499,504 adults from 31 to 69 years-old observed that current smokers were less likely to be obese than never smokers (OR = 0.83; 95% CI: 0.81, 0.86) and former smokers were more likely to be obese than both current smokers (OR = 1.33; 95% CI: 1.30, 1.37) and never smokers (OR = 1.14; 95% CI: 1.12, 1.15), however this association varied according to age, sex, and amount smoked [11]. Several factors may be related to this association, such as the belief that smoking is an effective way of reducing body weight, change in food preference, lower dietary energy intake, higher energy expenditure, or modifications in the metabolism of calories [11]. Between 1990 and 2015, Brazil recorded a sustained progress in tobacco control, the single most important preventable factor for death and illness, with a reduction of 56% in smoking prevalence [50, 51]. However, despite the increasing frequency of non-smokers observed from 2003 to 2015 in the present study, Sao Paulo remains one of the State capitals in the country with the highest tobacco use [17]. Our results show that, compared to non-smokers, current smokers were less likely to have excess body weight and former smokers presented 46% more chances of having excess body weight and obesity. Even though one of the main reasons cited for not trying to quit smoking is fear of weight gain, research shows that smoking cessation is associated with substantial health benefits, including improved insulin sensitivity even in the presence of weight gain [51]. Thus, policies and interventions focused on both smoking and diet could increase success rate in terms of smoking cessation and prevent weight gain.

Regarding socioeconomic status, excess body weight has increased at all levels of income during the past decades. Although the annual incremental rates indicate an increase in the incidence of obesity among the poorest men and women in Brazil, those with higher income present the highest rates, especially among men [15]. In the city of Sao Paulo, those who earn more than one minimum wage per person per month in the household are 14% more likely to have excess body weight and obesity. These findings may be associated with factors such as food environment, access and food security, as well as built environment and movement from physical to sedentary labor [10, 52]. In addition, studies have shown that education is inversely associated with excess body weight [9], but in the present study, this association was not statistically significant. Worldwide, the association between socioeconomic status and body weight is dependent on the level of economic development of the country [10]. Generally, the prevalence of obesity is positively associated with the initial stages of economic growth, as populations go through nutritional and lifestyle transitions with little access to education and health services. As income increases, some habits associated with obesity are adopted, such as television viewing, purchasing and consuming more fast food, convenience foods, and other high-energy and low-quality foods. However, when there is improved access to health services, education, exercise, and healthy food, as associated with behavioral changes, this association declines. Nevertheless, those factors remain limited. In Brazil, only the quartile of women with the highest income has a lower prevalence of overweight than the quartile with the lowest income [53].

Another social factor that has an important role in lifestyle and is also associated to excess body weight is marital status. In the present study, being single significantly reduced the chances of being overweight or obese compared to people who are married or have a common law partner. Similar results were observed in other populations, which had high rates of overweight or obesity relative to adults in other marital status groups, particularly among men [54–56]. On the other hand, research indicates that married adults were generally found to be healthier than adults in other marital status categories [55].

Taking these factors into account, the prediction models for excess body weight and obesity indicate that an important increase in their prevalence will occur in all age groups if the patterns observed in the latest surveys do not change. Secular trends estimate that 38% of adults will be overweight and 20% will be obese worldwide by 2030 [57]. As many aspects influence weight gain and the changes in environment in the population level are dynamic, the trends observed in this study may accelerate, stop, or slow. At the same time that some countries have been observing a slowing of increases in obesity prevalence [58, 59], others have reached extreme values, such as American Samoa, in Pacific Islands, where 75% of the general population is considered obese [60]. In USA, projections indicate that over 85% of adults will have excess body weight by 2030 [61]. Therefore, due to the rapid increase in prevalence of excess body weight in Sao Paulo observed in this 12-year period and the existing uncertainties when making predictions based on past data [62, 63], we highlight the need for continuous surveillance in order to enable the identification, implementation, and evaluation of evidence-based actions to face this public health problem.

Some limitations should be considered in interpreting the findings of the current study. First, the values for height and weight used to calculate the body mass index were self-reported by the individuals during the household interview, which could lead to underestimates of the population prevalence of overweight and obesity, especially if there is a propensity of over-reporting height and/or under-reporting weight. Although self-reported data may be subject to inaccuracies; they were validated in previous study with ISA-Capital population [30], which observed high intraclass correlation between self-reported and measured parameters for weight (*r* > 0,94) and BMI (r > 0,85). The agreement between measured and self-reported weight, height and BMI was good, as sensitivity was > 91% and specificity was > 83%. In general, the use of self-reported lifestyle habit variables is also vulnerable to biases, but many precautions were taken in the study design to minimize possible errors, such as the face-to-face interview held in the households, selection of qualified interviewers and training according to the standard operating procedures, besides periodical meetings between the interviewers and the coordinating staff to check if the procedures were being used accurately and to discuss doubts or potential problems. A second limitation is that we did not explore in detail the patterns of income during this period, but the profound economic crises that Brazil passed through the year of 2015 [64] may have had important influence on this association, as we observed an increase in the prevalence of people in the lowest income category in 2015. There is evidence that changes in socioeconomic position across life course influence excess body weight [65, 66], but future research is needed to explore this association in different contexts. A third limitation, is that due to the fact that overweight and obesity have multifactorial causes, many factors that could be associated to it, such as diet quality, energy intake, sleeping habits, sedentary behavior, psychosocial factors, gut microbiome, in-utero and physical environment, media and marketing exposure, and genetic and epigenetic variations [13, 14], could not be assessed in this analysis; however, as a complex issue, the association of evidences from multiple researches may help to elucidate this public health challenge. Finally, although we discussed many possible causes for the increases in obesity prevalence, it is important to observe that the cross-sectional design of the study precludes causal statements. Still, the survey design is adequate to properly answer the proposed objectives in this analysis: identification of individuals with a higher likelihood of disease occurrence for public health purposes [67].

Despite of the limitations, the present study represents the largest investigation of overweight and obesity in the city of São Paulo, with multiple time points and a sampling design that represents all the population aged 12 years and older living in households in the urban area of the city.

## Conclusion

Our findings present up-to-date information about the distribution of excess body weight, which increased substantially over a short time and more prominently in specific groups of the population, such as female adolescents and adults. The factors associated with excess body weight, such as gender, income, age group, and smoking status, may provide important information for decision makers and researchers to create or review the existing programs and interventions in order to decrease the trend for the next years.

## Abbreviations

AIC: Akaike’s Information Criterion
BIC: Basic Information Criterion
BMI: Body Mass Index
CI: Confidence Interval
DALYs: Disability-Adjusted Life Years
EBW: Excess Body Weight
ISA-Capital: *Inquérito de Saúde de São Paulo*, Health Survey of São Paulo
OPAS: Pan American Health Organization
OR: Odds Ratio
SABE: Health, Well-Being, and Aging Study
SD: Standard Deviation
VIGITEL: Surveillance of Risk and Protective Factors for Chronic Diseases by Telephone Survey
